# Author Correction: A potential explanation for the global increase in tropical cyclone rapid intensification

**DOI:** 10.1038/s41467-022-35497-7

**Published:** 2022-12-23

**Authors:** Kieran Bhatia, Alexander Baker, Wenchang Yang, Gabriel Vecchi, Thomas Knutson, Hiroyuki Murakami, James Kossin, Kevin Hodges, Keith Dixon, Benjamin Bronselaer, Carolyn Whitlock

**Affiliations:** 1 Guy Carpenter, New York, NY USA; 2 National Centre for Atmospheric Science and Department of Meteorology, University of Reading, Reading, Berkshire, UK; 3 Department of Geosciences, Princeton University, Princeton, NJ USA; 4 High Meadows Environmental Institute, Princeton University, Princeton, NJ USA; 5 NOAA/Geophysical Fluid Dynamics Laboratory, Princeton, NJ USA; 6 The Climate Service, an S&P Global company, Madison, WI USA; 7 Englehart Commodities Trading Partners, London, UK; 8 NOAA/Geophysical Fluid Dynamics Laboratory, Princeton, and Engility Inc., Dover, NJ USA

**Keywords:** Atmospheric dynamics, Natural hazards, Attribution, Climate and Earth system modelling

Correction to: *Nature Communications* 10.1038/s41467-022-34321-6, published online 04 November 2022

The original version of this Article contained errors in the y-axis of Fig. 3a and 3b, in which Fig. 3a had an incorrect title in the y-axis, and fig. 3b had incorrect numbers in the y-axis. This has been corrected in both the PDF and HTML versions of the Article.

