# Author Correction: A potential explanation for the global increase in tropical cyclone rapid intensification

**DOI:** 10.1038/s41467-023-36308-3

**Published:** 2023-01-31

**Authors:** Kieran Bhatia, Alexander Baker, Wenchang Yang, Gabriel Vecchi, Thomas Knutson, Hiroyuki Murakami, James Kossin, Kevin Hodges, Keith Dixon, Benjamin Bronselaer, Carolyn Whitlock

**Affiliations:** 1 Guy Carpenter, New York, NY USA; 2 National Centre for Atmospheric Science and Department of Meteorology, University of Reading, Reading, Berkshire, UK; 3 Department of Geosciences, Princeton University, Princeton, NJ USA; 4 High Meadows Environmental Institute, Princeton University, Princeton, NJ USA; 5 NOAA/Geophysical Fluid Dynamics Laboratory, Princeton, NJ USA; 6 The Climate Service, an S&P Global company, Madison, WI USA; 7 Englehart Commodities Trading Partners, London, UK; 8 NOAA/Geophysical Fluid Dynamics Laboratory, Princeton, and Engility Inc., Dover, NJ USA

**Keywords:** Atmospheric dynamics, Natural hazards, Attribution, Climate and Earth system modelling

Correction to: *Nature Communications* 10.1038/s41467-022-34321-6, published online 04 November 2022

The original version of this Article contained an error in the caption of Figure 3a, which incorrectly read ‘showing ratios of rapid intensification (RI) cases satisfying the critical environmental thresholds divided by entire samples satisfying thresholds (blue histograms) and the analogous ratios for cases where the critical thresholds are not met (red histograms)’. The correct version replaces sentence with ‘showing the probability of rapid intensification (RI) for cases satisfying the critical environmental thresholds (blue histograms) and the probability of RI for cases where the critical thresholds are not met (red histograms)’. This has been corrected in both the PDF and HTML versions of the Article.

