# Supplementary material for: A potential explanation for the global increase in tropical cyclone rapid intensification
Source: Nat Commun. 2022 Nov 4;13:6626. doi: 10.1038/s41467-022-34321-6 (PMC9636401; doi:10.1038/s41467-022-34321-6)
Supplement: Supplementary file 1 — Supplementary Information [file 41467_2022_34321_MOESM1_ESM.pdf]

<u>A Potential Explanation for the Global Increase in Tropical Cyclone Rapid Intensification</u>

Kieran Bhatia[1*]
Alexander Baker[2]
Wenchang Yang[3]
Gabriel Vecchi[34]
Thomas Knutson[5]
Hiroyuki Murakami[5]
James Kossin[6]
Kevin Hodges [2]
Keith Dixon[5]
Benjamin Bronselaer[7]
Carolyn Whitlock[8]

[1]Guy Carpenter, New York, NY, USA
[2]National Centre for Atmospheric Science and Department of Meteorology, University of Reading, Reading, Berkshire, UK
[3]Department of Geosciences, Princeton University, Princeton, New Jersey, USA
[4]High Meadows Environmental Institute, Princeton University, Princeton, New Jersey, USA
[5]NOAA/Geophysical Fluid Dynamics Laboratory, Princeton, New Jersey, USA
[6] The Climate Service, an S&P Global company, Madison, WI, USA
[7]Englehart Commodities Trading Partners, London, UK
[8]NOAA/Geophysical Fluid Dynamics Laboratory, Princeton, and Engility Inc., Dover, New Jersey, USA
[*]Correspondence should be addressed to Kieran T. Bhatia, Guy Carpenter, 1166 6th Ave, 32nd floor, New York, NY, USA 10036, email: Kieran.bhatia@gmail.com

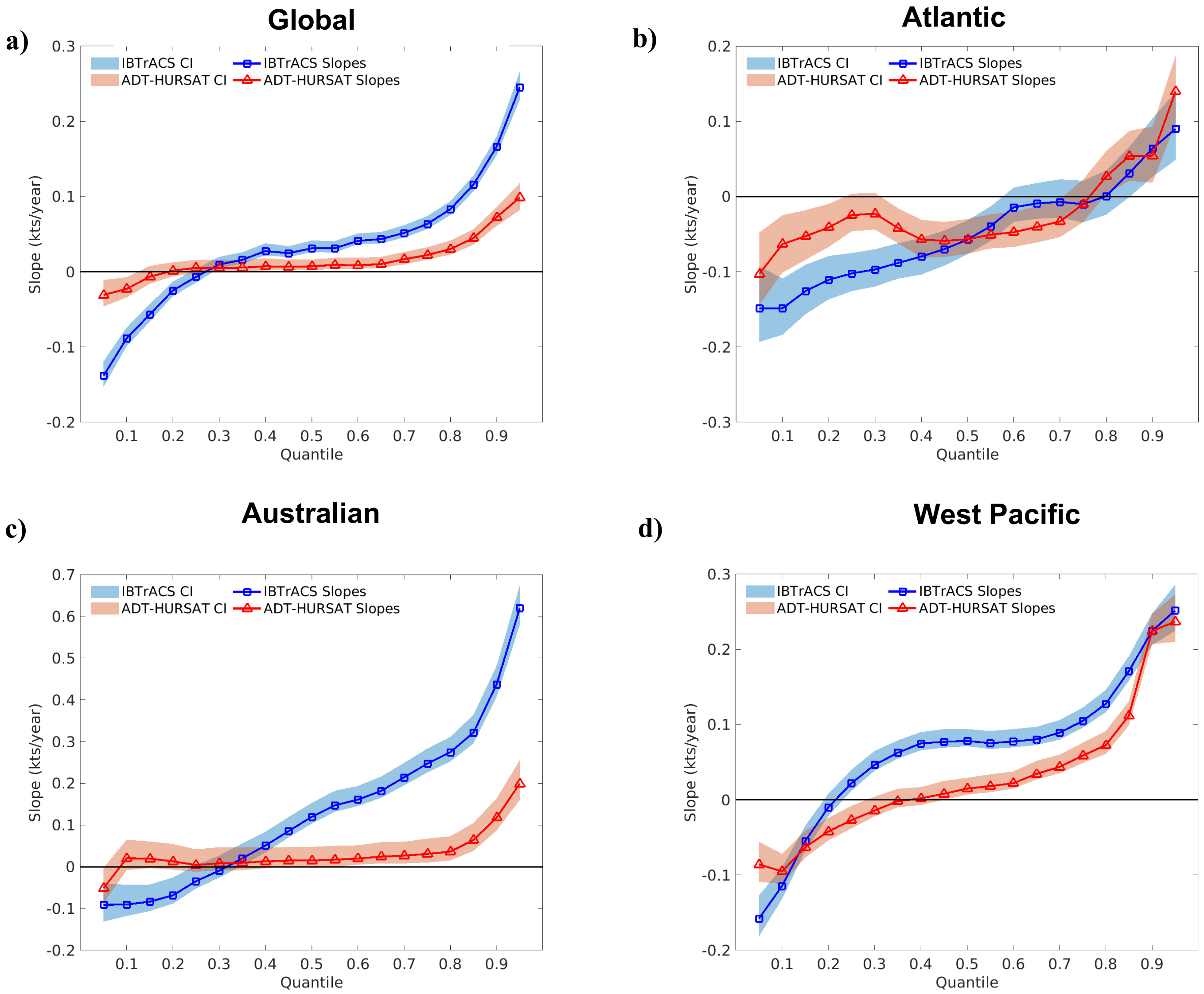

Supplementary Fig. 1: Quantile Regression

Quantile regression of 24-h intensity changes. Slope of the quantiles for 24-h intensity changes during the period 1982–2017 in four basins: (a) Global, (b) Atlantic, (c) Australian, (d) West Pacific. (a–d) Slopes are shown for IBTrACS (blue) and ADT-HURSAT (red). The squares and triangles represent the slope derived from least squares regression of intensity change as a function of year for each quantile from 0.05 to 0.95 in steps of 0.05. Shading represents the 5th and 95th percentiles of the regressions with randomly perturbed observational data (Methods).

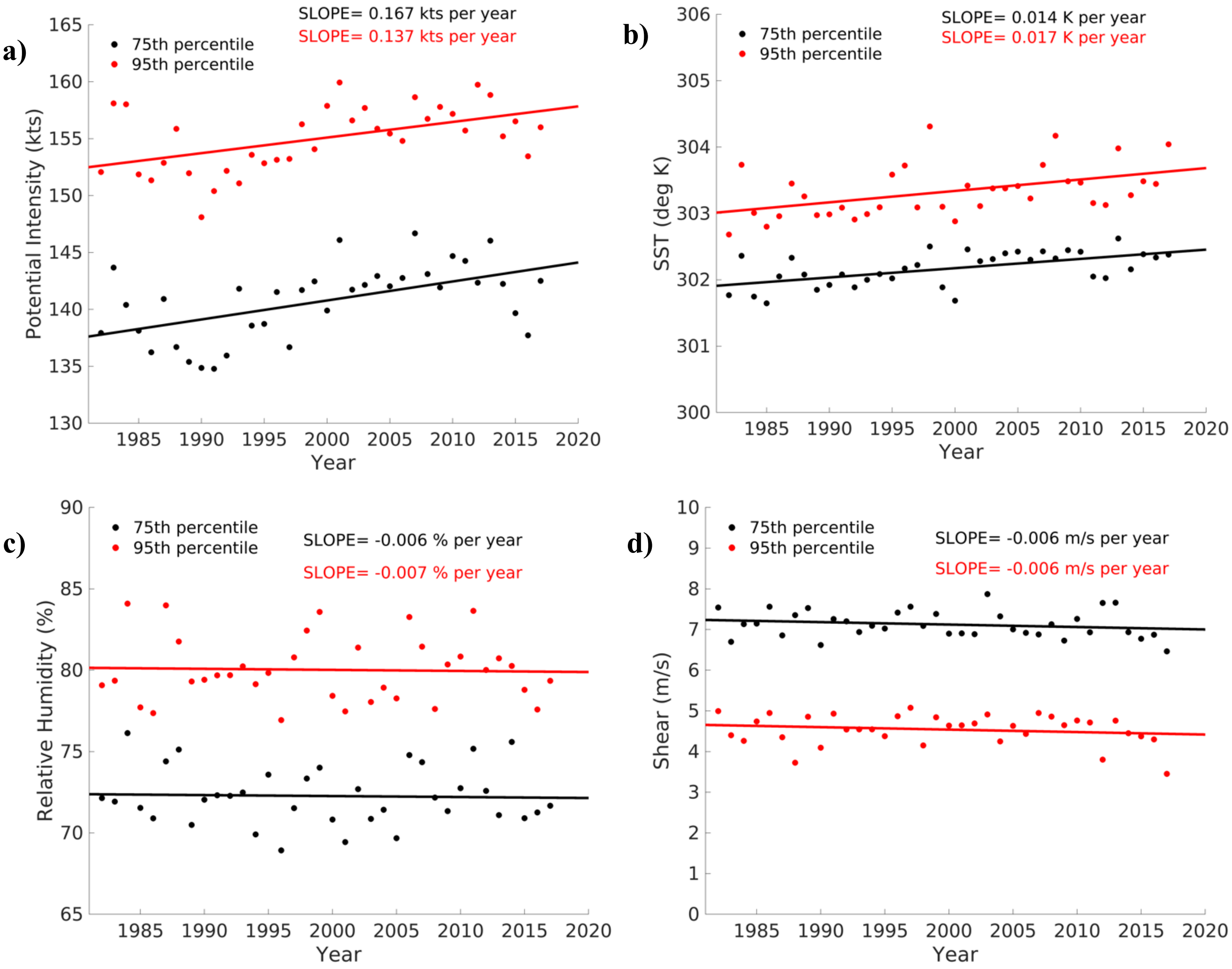

Supplementary Fig. 2: Storm-Local Environment Trends

a-d Observed trends in the 75th (black) and 95th percentile (red) of ERA5 storm-local environments over the 36-year period 1982–2017 using (a) potential intensity (PI) (b) sea surface temperature (SST) (c) relative humidity (RH) (d) vertical wind shear (SHR) data. The methodology used to calculate the storm-local environments is detailed in the Methods section. Annual values are denoted by dots, and the slope derived from least squares regression of annual values are included for each percentile and environmental parameter.

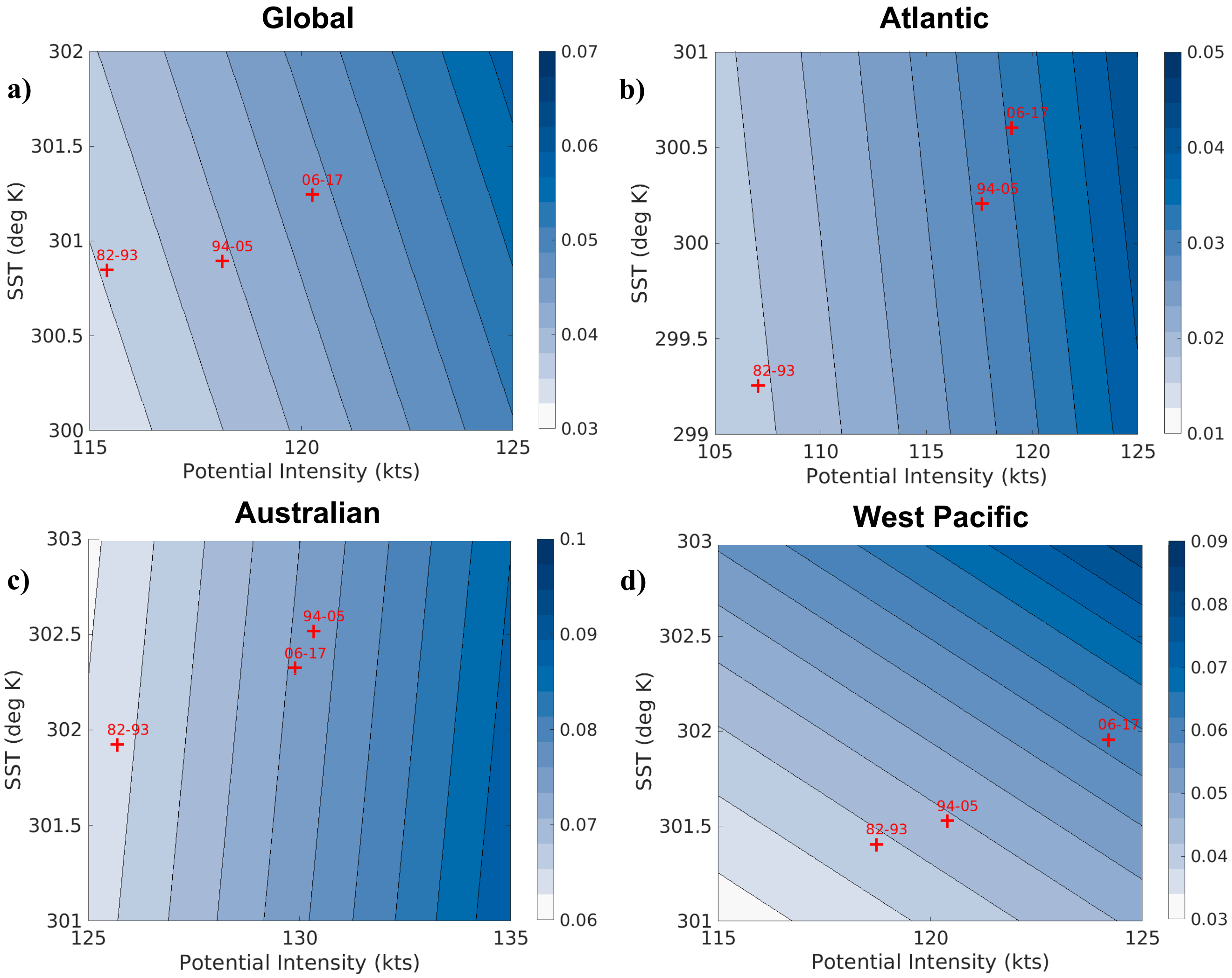

Supplementary Fig. 3: Time Evolution of the Probability of Rapid Intensification vs. Sea Surface Temperature and Potential Intensity

a-d The probability of IBTrACS rapid intensification (RI) in four regions: (a) Global, (b) Atlantic, (c) Australian, (d) West Pacific is contoured based on a logistic regression with two predictors, ERA5 sea surface temperature (SST) and potential intensity (PI). The contour shading represents the probability of RI with darker colors corresponding to higher probabilities of RI. The mean values of SST and PI for the first (1982-1993), second (1994-2005), and third (2006-2017) terciles are plotted as red plus signs.

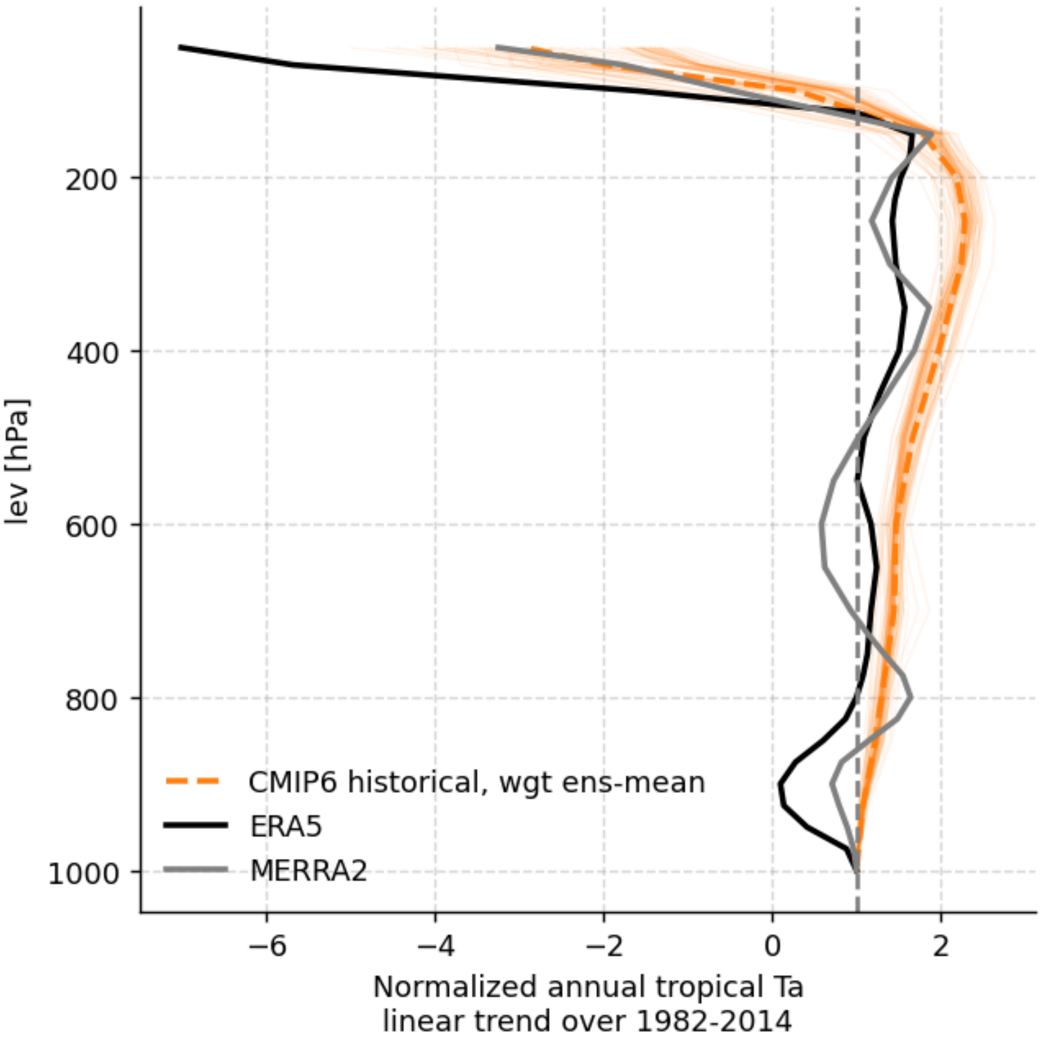

Supplementary Fig. 4: Vertical Profile of Tropical-Mean Linear Trends

Trends in the tropospheric vertical profile of annual mean air temperature (Ta) in ERA5 (black), MERRA2 (grey), and CMIP6 simulations (orange; ensemble mean is bolded, and ensemble members are dashed lines) over the period 1982–2014. Trends were computed for the ocean gridboxes between 30ºN–30ºS and heights ranging from 1000-50 hPa. Values for grid boxes were only included if potential intensity (PI) is available at the location for the historical, hist-nat and hist-GHG experiments. Vertical profiles were normalized by values at 1000 hPa.

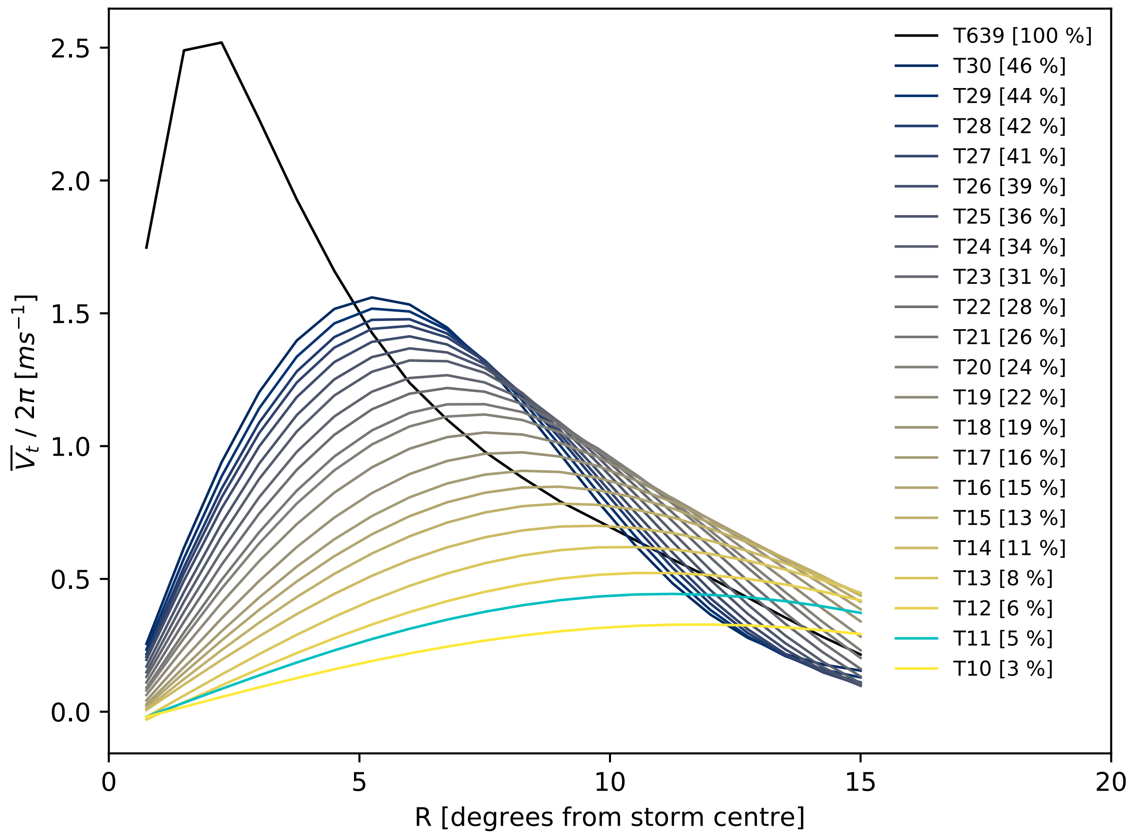

Supplementary Fig. 5: Spectral Filtering Sensitivity Testing

Tropical cyclone tangential wind (Vt) as a function of cyclone radius (R) for the full, native ERA5 resolution (T639) and for truncations in the range T30–T10. Vt was computed on a radial grid and composited for all tropical cyclones. The value in brackets gives the percentage of the T639 circulation remaining in the spectrally filtered field, estimated by taking the difference between the T639 and filtered fields, integrated between 0–5º. T11 was used to prepare storm-local environments because it removed 95% of the composited tropical cyclones' circulations.

Variable/Basin	AL	EP	WP	SP	AU	GL
RH	60.4%	71.9%	67.8%	68.4%	67.8%	67.4%
SHR	9.2 m/s	7.9 m/s	9.5 m/s	10.9 m/s	10.1 m/s	9.3 m/s
SST	300.8 K	300.4 K	302.0 K	301.7 K	302.4 K	301.4 K
PI	129.0 kts	130.6 kts	132.4 kts	132.0 kts	133.6 kts	131.3 kts
RI	5.3%	8.5%	8.7%	7.1%	8.5%	7.7%

Supplementary Table 1: Critical Environmental Thresholds for Each Basin

Logistic regression is used to determine critical ERA5 environment thresholds for each basin and environmental parameter. The variable “RI” corresponds to the percentage of 24-hour IBTrACS intensity changes that exceed 30 knots in each basin. A critical threshold is defined as the environmental parameter value that yields the mean probability of a 24-hr wind speed exceeding 30 knots between 1982-2017.

Thresholds Met/Basins	AL	EP	WP	SP	AU	GL
0	9/715	9/627	22/1873	6/540	9/427	41/4035
1	22/1030	51/1617	108/2992	10/560	36/677	207/7082
2	77/1404	121/1541	272/3425	37/498	85/862	588/8340
3	91/1050	176/1082	408/2617	52/390	78/590	862/6222
4	40/306	83/326	225/948	53/248	22/152	428/1925
Total Cases	239/4505	440/5193	1035/11855	158/2236	230/2708	2126/27604

Supplementary Table 2: Rapid Intensification Ratios for Different Critical Thresholds

Table shows the number of times that the ERA5 storm-local environmental parameters satisfied each number of critical thresholds (second number) and the number of observed rapid intensification (RI) events (first number) for these situations.

Sea Surface Temperature		Potential Intensity		Relative Humidity		Wind Shear	
Model	Ensemble Members (Total=101 members)	Model	Ensemble Members (Total= 87 Members)	Model	Ensemble Members (Total= 97 Members)	Model	Ensemble Members (Total= 93 Members)
ACCESS-CM2	3	ACCESS-CM2	3	ACCESS-CM2	3	ACCESS-CM2	1
ACCESS-ESM1-5	3	ACCESS-ESM1-5	3	ACCESS-ESM1-5	3	ACCESS-ESM1-5	3
BCC-CSM2-MR	3	BCC-CSM2-MR	3	BCC-CSM2-MR	3	BCC-CSM2-MR	3
CESM2	3	CESM2	3	CESM2	1	CESM2	3
CNRM-CM6-1	10	CNRM-CM6-1	9	CNRM-CM6-1	10	CNRM-CM6-1	10
CanESM5	46	CanESM5	41	CanESM5	45	CanESM5	38
FGOALS-g3	1	FGOALS-g3	1	FGOALS-g3	3	FGOALS-g3	3
GFDL-ESM4	1	GFDL-ESM4	1	GISS-E2-1-G	5	GFDL-ESM4	1
GISS-E2-1-G	5	GISS-E2-1-G	5	HadGEM3-GC31-LL	5	GISS-E2-1-G	5
HadGEM3-GC31-LL	5	HadGEM3-GC31-LL	5	IPSL-CM6A-LR	10	HadGEM3-GC31-LL	5
IPSL-CM6A-LR	10	IPSL-CM6A-LR	10	MIROC6	3	IPSL-CM6A-LR	10
MIROC6	3	MIROC6	3	MRI-ESM2-0	5	MIROC6	3
MRI-ESM2-0	5			NorESM2-LM	1	MRI-ESM2-0	5
NorESM2-LM	3					NorESM2-LM	3

Supplementary Table 3: CMIP6 Models and Ensemble Members for Analysis

Table shows the CMIP6 models and ensemble members used for tropical-mean comparisons for each environmental variable. To be included in the sample, each model required an ensemble member for historical, hist-nat, and hist-GHG simulations.
